# Wait and consult times for primary healthcare services in central Mozambique: a time-motion study

**DOI:** 10.3402/gha.v9.31980

**Published:** 2016-08-30

**Authors:** Bradley H. Wagenaar, Sarah Gimbel, Roxanne Hoek, James Pfeiffer, Cathy Michel, Fatima Cuembelo, Titos Quembo, Pires Afonso, Stephen Gloyd, Barrot H. Lambdin, Mark A. Micek, Victoria Porthé, Kenneth Sherr

**Affiliations:** 1 Department of Global Health, University of Washington School of Public Health, Seattle, WA, USA; 2 Health Alliance International, Seattle, WA, USA; 3 Department of Family Child Nursing, University of Washington, Seattle, WA, USA; 4 Health Alliance International, Beira, Mozambique; 5 Beira Operations Research Center, Ministry of Health, Beira, Mozambique; 6 Community Health Department, School of Medicine, Eduardo Mondlane University, Maputo, Mozambique; 7 Pangea Global AIDS, Oakland, CA, USA

**Keywords:** health services research, maternal and child health, maternity services, primary health care, priority setting, healthcare allocation, health professionals

## Abstract

**Background:**

We describe wait and consult times across public-sector clinics and identify health facility determinants of wait and consult times.

**Design:**

We observed 8,102 patient arrivals and departures from clinical service areas across 12 public-sector clinics in Sofala and Manica Provinces between January and April 2011. Negative binomial generalized estimating equations were used to model associated health facility factors.

**Results:**

Mean wait times (in minutes) were: 26.1 for reception; 43.5 for outpatient consults; 58.8 for antenatal visits; 16.2 for well-child visits; 8.0 for pharmacy; and 15.6 for laboratory. Mean consultation times (in minutes) were: 5.3 for outpatient consults; 9.4 for antenatal visits; and 2.3 for well-child visits. Over 70% (884/1,248) of patients arrived at the clinic to begin queuing for general reception prior to 10:30 am. Facilities with more institutional births had significantly longer wait times for general reception, antenatal visits, and well-child visits. Clinics in rural areas had especially shorter wait times for well-child visits. Outpatient consultations were significantly longer at the smallest health facilities, followed by rural hospitals, tertiary/quaternary facilities, compared with Type 1 rural health centers.

**Discussion:**

The average outpatient consult in Central Mozambique lasts 5 min, following over 40 min of waiting, not including time to register at most clinics. Wait times for first antenatal visits are even longer at almost 1 h. Urgent investments in public-sector human resources for health alongside innovative operational research are needed to increase consult times, decrease wait times, and improve health system responsiveness.

## Introduction

Since the 2006 World Health Report, which highlighted the global crisis of a shortage of 4.3 million trained health workers globally, research and policy attention has increased around human resources for health (HRH) in low- and middle-income countries (LMICs) (1). Mozambique has an estimated 0.03 doctors and 0.21 nurses per 1,000 population, which remains significantly below the absolute minimum World Health Organization (WHO) targets of 2.5 doctors, nurses, and midwives per 1,000 population (1, 2). Significant efforts are underway to decrease this HRH gap in LMICs, yet the African region alone is estimated to have a $2.6 billion annual wage gap ($1.1 billion for doctors and $1.5 billion for nurses and midwives) (3). Mozambique has been a leader in task-sharing for almost two decades, through the development of rapid training and re-training programs to certify lower level providers to supplement care provided by limited doctors and specialists. Specifically, the country rolled-out a non-physician clinician (*técnicos de medicina*) program in 2004 to rapidly expand ART care (4, 5) and has been training psychiatric technicians (*técnicos de psiquiatria*) to provide the backbone of mental healthcare identification and treatment since 1996 (6).

With expanding availability of newer health interventions such as Option B+ (immediate initiation of lifelong ART for pregnant and breastfeeding women with HIV) for the prevention of mother-to-child transmission of HIV (PMTCT) (7), new diagnostic and treatment guidelines for tuberculosis and malaria (8, 9), pneumococcal conjugate and rotavirus vaccines for well-child care (10), and the integration of mental health services into primary health care, more and more patients are seeking care in outpatient facilities outstripping the public-sector health workforce capacity of many developing countries. Recruitment and training of more providers and improvements in infrastructure take time and resources which are currently unavailable. Even if Mozambique reaches its goal to double the number of nurses by 2015, rapid population growth, low absolute numbers of nurses, and attrition mean it will take sustained investments over a decade or more to reach even minimum WHO targets around HRH. In the meantime, increasing the efficiency of existing health facilities, through examination of health facility-level predictors of patient wait and consult time using time-motion studies to target policy and strategy response, is one way to meet the growing needs of sick populations in resource-limited settings.

Time-motion studies emerged in the early 1900s from engineering and industrial settings focused on reducing inefficiencies in human effort and materials during the production process (11). The idea of a time-motion study is to have external observers capture data continuously on a system in order to identify bottlenecks and inefficiencies to improve system performance. Most previous time-motion studies in sub-Saharan African settings have focused on shadowing how health workers spend their time in clinical settings (12–18). Few studies have focused on tracking progression through clinic visits from a patient perspective.

Time-motion studies that document patient wait and consult times are able to highlight the experience of patients during health facility visits to subsequently inform recommendations for changes around staffing allocation, infrastructure improvements, adaptations to workplace norms and culture, and to evaluate effects of new programs using cost-effectiveness analyses. Past studies have demonstrated that increased waiting time for patients has adverse effects on health-seeking behavior, patient satisfaction, and treatment adherence, whereas increased consultation time has positive effects (19, 20). Previous research in Mozambique has identified short waiting times at public-sector health clinics in Manica Province as the factor most strongly related to patient satisfaction (21). Globally, patient satisfaction has been associated with increased utilization of health services (22–24), and satisfied patients are also more likely to follow clinician directions and return for follow-up care (25, 26).

The purpose of the present study was to 1) describe wait and consult times across a comprehensive list of services provided at public-sector primary and higher level clinics and 2) identify health facility determinants of shorter wait times and longer consult times. These data may be of interest to Ministries of Health, health workers, and the global research community as we work to provide the highest quality of health care to populations in LMICs while constrained by ongoing shortages of trained health workers.

## Methods

### Study design

Cross-sectional study as part of a preintervention baseline assessment.

### Study setting

Twelve public-sector health facilities (six from Sofala Province and six from Manica Province) were purposefully selected to reflect a spectrum of public-sector health facilities with regard to size, health worker staffing patterns, types of services offered, and utilization patterns. Manica serves as the control province to a 7-year comprehensive primary health system intervention underway in Sofala Province (27). Manica was selected because it is similar in terms of population size (approximately 1.7–1.9 million) (28), number of districts, baseline health measures, and culture. Under-5 mortality was estimated as 114 in Manica and 105 in Sofala per 1,000 live births for the year 2011 (29). Health workforce density for the years 2006–2010 was 53.3 per 100,000 population in Manica and 70.6 in Sofala; population per public-sector health facility for the same years was 16,322 in Manica and 11,638 in Sofala (30).

### Data collection

**Fig. 1:**
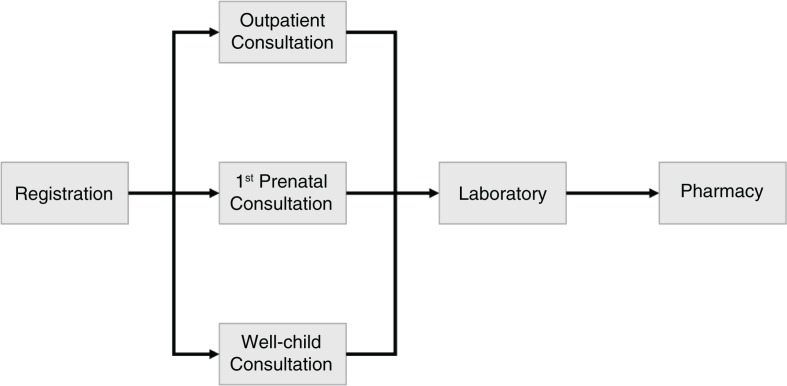
Flow map of process for different services observed in time-motion study, January–April 2011, Central Mozambique.

Wait and consult times were assessed through direct observation of patients as they passed through services available at each health facility (general reception, outpatient consults, antenatal visits, well-child visits, pharmacy, and laboratory) from January to April 2011. Observation was conducted by research assistants who were stationed at each step of care, from registration (when a patient gets in line to pay the one metical registration fee [$USD=3 cents] for an outpatient consultation) to outpatient consultations, and then to laboratory and/or pharmacy (Fig. 1). Wait and consult times for first antenatal visits and well-child visits were also observed. To clarify, we did not follow individual patients through all phases of the clinical encounter, but instead recorded the time of all patients who sequentially arrived and left each clinical service area. Research assistants observed patients at their selected service during normal business hours (7:30 am to 3:30 pm), or when daily service delivery was completed. Research assistants observed patients at each health facility for 4 days – anticipated to allow the observation of a minimum of 160 patients across care pathways. A minimum of 80 patients were targeted to be observed at the two rural hospitals due to smaller patient loads (Nhansato and Mude Type 2 Rural Health Centers). Research assistants consisted of six data collectors and one supervisor at each health facility, working out at the Beira Operations Research Center. All data collectors and supervisors attended a multiple-day training on basic research design and analysis, as well as the specifics of the present study.

Waiting times for registration were determined based on the time patients spent since arriving at the reception (formed a queue) until they were met by the receptionist. Consultation waiting times were determined based on the time patients spent waiting at the door of the service until they were met by the provider. Similar procedures were used to operationalize pharmacy and laboratory waiting times. Consultation times were determined by the time patients entered the consultation room or area until the patient left. Patients who left the facility and were lost to follow-up were excluded from the analyses.

### Data management, variable descriptions, and analyses

Paper data forms were transported to the Beira Operations Research Center, where data were double-entered into Microsoft Excel, finalized, and cleaned. Our analysis plan included: 1) descriptive statistics of the 12 health facilities surveyed; 2) descriptive statistics of wait and consult times by primary healthcare service provision and by available health facility predictors; and 3) bivariate negative binomial regression models controlling for facility clustering using generalized estimating equations and robust standard errors to evaluate predictors of wait times and consult times. We used Stata 13 (StataCorp., College Station, TX) for statistical analyses. Associations were assessed for statistical significance at *α*=0.05 using two-tailed tests.

Available predictors with complete data availability for descriptive statistics and regression modelling included: type of health facility (see Table 1), rural/urban clinic location, number of outpatient consults in 2010, and number of institutional births in 2010. The number of consults and institutional births by clinic was abstracted from the National Health Information System (*Módulo Básico*)**. These predictors were selected to allow for analyses of wait and consult time by programmatically relevant factors (type of health facility and rural/urban location), as well as proxies for patient volume and workload for the most recent complete year of data collection (outpatient consults and institutional births in 2010). The Mozambican health system is organized into four levels (primary, secondary, tertiary, and quaternary), with nine types of health facilities. See Table 1 for a detailed description of the different types of health facilities.

**Table 1:** List of types of health facilities, technical characterizations, and list of specific functions of National Health Service Institutions as classified by the Mozambican Ministry of Health

Type of health facility	Catchment area size	Typical location	Human resources	Outpatient services	Prenatal services	Well-child services	Inpatient services	Maternity services	Laboratory	Pharmacy
Rural health centres										
Type 1	16,000–35,000	District headquarters	13–16 staff	Yes	Yes	Yes	Yes	Yes	Yes	Yes
Type 2	7,500–20,000	Rural gathering point	4 staff	Yes	Yes	Yes	No	Yes	No	No
Urban health centres										
Type A	40,000–100,000	High-density city area	26–36 staff	Yes	Yes	Yes	No	Yes	Yes	Yes
Type B	18,000–48,000	High-density city area	14 staff	Yes	Yes	Yes	No	No	Yes	Yes
Type C	10,000–25,000	Less-populated city area	4 staff	Yes	Yes	Yes	No	No	No	Yes
Hospitals										
Central	>2,000,000	Selected cities nationally	Not specified	Yes	No	No	Yes	Yes	Yes	Yes
Provincial	800,000–2,000,000	Provincial capital	Not specified	Yes	No	No	Yes	Yes	Yes	Yes
Rural/general	150,000–900,000	District headquarters	61–102 staff	Yes	Yes	Yes	Yes	Yes	Yes	Yes
District	50,000–250,000	District headquarters	32–42 staff	Yes	Yes	Yes	Yes	Yes	Yes	Yes

## Results

In total, 8,102 patients were observed. Clinics included in the sampling frame conducted a median of 74,082 outpatient consultations (range: 4,576–228,588) in 2010 and 2,331 institutional births (range: 152–6,022). Eight of the 12 facilities were located in rural areas, and two clinics were observed across each of the five different classifications of health facilities included in this study, with the exception of Type 1 rural health centers, where four facilities were selected for patient observation (see Table 2). Of those patients observed at general reception (*N*=1,248), 20.9% (261) arrived before 8:30 am, 26.9% (336) between 8:30 and 9:30 am, 23.0% (287) between 9:30 and 10:30 am, 17.0% (212) between 10:30 and 11:30 am, 7.9% (99) between 11:30 am and 12:30 pm, and 4.2% (53) after 12:30 pm. A small number of patients (*n*=25; 0.3%) were lost to follow-up and were excluded from all analyses.

**Table 2:** Characteristics of 12 health facilities surveyed and 8,102 patients observed through time-motion study in central Mozambique, January–April, 2011

Characteristic	Number of clinics, *n* (%) unless noted	Number patients observed at clinics, *n* (%) unless noted
Total	12 (100)	8,102 (100)
Outpatient consults in 2010, mean (SD)	88,587 (62,993)	N/A
Institutional births in 2010, mean (SD)	2,604 (1,844)	N/A
Rural clinic location	8 (66.7)	4,407 (54.4)
Type of health facility		
Provincial/central hospital	2 (16.7)	2,105 (26.0)
Urban health center – Type A	2 (16.7)	1,590 (19.6)
Rural hospital	2 (16.7)	1,338 (16.5)
Rural health center – Type 2	2 (16.7)	430 (5.3)
Rural health center – Type 1	4 (33.3)	2,639 (32.6)

N/A, not applicable because all clinics are included in sample.

### Average wait and consultation times across service provision areas

Mean wait and consultation times across service provision are presented in Table 3. Mean wait times in minutes were longest for first antenatal visits (58.8), followed by outpatient consults (43.5) and general registration (26.1). The 90th percentile of wait times showed patients waiting over an hour for general registration, over an hour and a half for outpatient consultation, and almost 2 h for first antenatal visits. Mean consultation times were longest for first antenatal visits at 9.4 min, followed by outpatient consults at 5.3 min, and well-child visits at 2.3 min. The 90th percentile of consult times is still very short, at 15 min for first antenatal visits, 10 min for outpatient consults, and 5 min for well-child visits.

**Table 3:** Wait and consult times by primary healthcare service provision, central Mozambique, January–April 2011

Service provided	*N* observed	Mean (95% CI)	Median	10th percentile	90th percentile
Registration					
Wait time	1,248	26.1 (24.3, 27.8)	12	1	76
Outpatient consults					
Wait time	1,011	43.5 (40.9, 46.0)	33	4	99
Consult time	1,373	5.3 (5.1, 5.6)	4	2	10
First prenatal visits					
Wait time	307	58.8 (54.3, 63.2)	54	14	112
Consult time	293	9.4 (8.9, 9.9)	9	4	15
Well-child visits					
Wait time	587	16.2 (14.0, 18.5)	5	1	46
Consult time	537	2.3 (2.1, 2.5)	1	0	5
Pharmacy					
Wait time	1,067	8.0 (7.3, 8.7)	4	1	20
Laboratory					
Wait time	1,679	15.6 (14.7, 16.6)	9	1	37

### Association between waiting times and health facility factors across service provision areas

Patients attending health facilities with more institutional births in 2010 (1,000 births change) had significantly longer wait times for general reception (RR: 1.4; 95% confidence interval [CI]: 1.1, 1.8), first antenatal visits (RR: 1.4; CI: 1.1, 1.9), and well-child visits (RR: 1.9; CI: 1.2, 3.0). Patients at Type A Urban Health Centers had significantly elevated wait times for outpatient consults (RR: 2.3; CI: 1.2, 4.7) compared with Type 1 rural health centers. Patients attending the smallest health facilities (Type 2 rural health centers) had significantly shorter wait times for first antenatal visits (RR: 0.55, CI: 0.30, 1.0), compared with Type 1 rural health centers (Table 4). Patients at both Type A urban health centers and rural hospitals had around five times (*p*<0.01) the wait times for well-child visits, compared with Type 1 rural health centers (Table 5). Similar to these results, patients attending clinics in a rural area had significantly shorter wait times for well-child visits (RR: 0.38; CI: 0.15, 0.98). Last, patients at rural hospitals were particularly poor performers regarding wait times around laboratory services, with 2.4 times (CI: 1.4, 4.1) the wait compared with Type 1 rural health centers.

**Table 4:** Predictors of wait times across general reception, outpatient consults, and first prenatal visits within 12 clinics in central Mozambique, January–April 2011

Characteristic	General reception wait time rate ratio (95% CI)	Mean (SD)	Outpatient consults wait time rate ratio (95% CI)	Mean (SD)	First prenatal visits wait time rate ratio (95% CI)	Mean (SD)
Total *N* patients observed	1,248		1,011		307	
Outpatient consults in 2010, per 10,000	1.1 (1.0, 1.1)	N/A	1.0 (0.99, 1.06)	N/A	1.04 (0.99, 1.09)	N/A
Institutional births in 2010, per 1,000	1.4** (1.1, 1.8)	N/A	1.1 (0.92, 1.23)	N/A	1.4* (1.08, 1.92)	N/A
Rural clinic location	0.68 (0.25, 1.9)	18.4 (26.6)	0.75 (0.44, 1.30)	36.3 (38.0)	0.58 (0.33, 1.0)	55.4 (40.8)
Type of health facility						
Provincial/central hospital	3.4 (0.68, 17.4)	38.7 (34.5)	1.5 (0.74, 3.0)	44.2 (41.3)	N/A	N/A
Urban health center – type A	2.0 (0.40, 10.4)	20.4 (25.0)	2.3* (1.2, 4.7)	69.4 (44.2)	1.9 (0.96, 3.6)	68.3 (35.6)
Rural hospital	3.1 (0.78, 12.5)	34.4 (31.9)	1.9 (0.64, 5.7)	55.6 (47.3)	1.8 (0.91, 3.6)	80.9 (41.7)
Rural health center – type 2	N/A	N/A	1.8 (0.65, 5.1)	46.1 (39.2)	0.55* (0.30, 1.0)	24.3 (14.0)
Rural health center – type 1	1 (reference)	7.9 (15.1)	1 (reference)	29.2 (32.2)	1 (reference)	45.3 (33.9)

* *p*<0.05

** *p*<0.01. N/A means this service does not exist at this factor/level (no patients observed).

**Table 5:** Predictors of wait times across well-child visits, pharmacy, and laboratory within 12 clinics in central Mozambique, January–April 2011

Characteristic	Well-child visits wait time rate ratio (95% CI)	Mean (SD)	Pharmacy wait time rate ratio (95% CI)	Mean (SD)	Laboratory wait time rate ratio (95% CI)	Mean (SD)
Total *N* patients observed	587		1,067		1,679	
Outpatient consults in 2010, per 10,000	1.1** (1.04, 1.17)	N/A	0.98 (0.92, 1.1)	N/A	0.99 (0.95, 1.03)	N/A
Institutional births in 2010, per 1,000	1.9** (1.18, 2.99)	N/A	0.97 (0.85, 1.1)	N/A	0.99 (0.87, 1.15)	N/A
Rural clinic location	0.38* (0.15, 0.98)	10.9 (23.4)	1.2 (0.60, 2.3)	8.8 (11.3)	1.2 (0.63, 2.4)	18.6 (23.4)
Type of health facility						
Provincial/central hospital	N/A	N/A	1.0 (0.59, 1.7)	6.1 (8.3)	1.0 (0.59, 1.7)	10.6 (13.9)
Urban health center – Type A	5.6** (3.3, 9.8)	31.5 (31.8)	1.5 (0.85, 2.5)	8.9 (15.6)	1.5 (0.85, 2.5)	17.1 (15.6)
Rural hospital	5.0** (1.8, 13.8)	25.3 (36.5)	2.4 (1.4, 4.1)	14.4 (15.7)	2.4** (1.4, 4.1)	26.6 (31.0)
Rural health center – Type 2	1.2 (0.22, 6.9)	2.6 (7.3)	N/A	N/A	N/A	N/A
Rural health center – Type 1	1 (reference)	4.9 (8.1)	1 (reference)	6.0 (7.0)	1 (reference)	12.3 (11.4)

* *p*<0.05

** *p*<0.01.

### Association between consult times and health facility factors across service provision areas

Compared with Type 1 rural health centers, patients attending Type 2 rural health centers (RR: 2.3; CI: 1.6, 3.4) had significantly longer outpatient consults, as did those at rural hospitals (RR: 1.7; CI: 1.4, 2.0) and tertiary- and quartenary-level facilities (RR: 1.4; CI: 1.2, 1.7) (Table 6). Compared with urban clinics, patients attending clinics located in rural areas had significantly longer well-child visits (RR: 1.7; CI: 1.2, 2.5). Patients at Type A Urban Health Centers had significantly shorter (RR: 0.56; CI: 0.44, 0.72) well-child consultation times compared with Type 1 rural health centers.

**Table 6:** Predictors of consult times across outpatient consults, first prenatal visits, and well-child visits within 12 clinics in central Mozambique, January–April 2011

Characteristic	Outpatient consults consult time rate ratio (95% CI)	Mean (SD)	1st Prenatal visits consult time rate ratio (95% CI)	Mean (SD)	Well-child visits consult time rate ratio (95% CI)	Mean (SD)
Total *N* patients observed	1,373		293		537	
Outpatient consults in 2010, per 10,000	0.98 (0.95, 1.0)	N/A	0.99 (0.98, 1.0)	N/A	0.98 (0.95, 1.01)	N/A
Institutional births in 2010, per 1,000	0.95 (0.85, 1.1)	N/A	1.0 (0.86, 1.17)	N/A	0.90 (0.65, 1.3)	N/A
Rural clinic location	1.3 (0.88, 1.9)	5.7 (5.4)	1.1 (0.82, 1.5)	9.7 (4.7)	1.7* (1.2, 2.5)	2.5 (2.6)
Type of health facility						
Provincial/central hospital	1.4* (1.2, 1.7)	5.6 (4.1)	N/A	N/A	N/A	N/A
Urban health center – Type A	0.91 (0.78, 1.1)	3.6 (1.7)	0.96 (0.72, 1.3)	8.8 (4.5)	0.56* (0.44, 0.72)	1.5 (1.3)
Rural hospital	1.7* (1.4, 2.0)	6.9 (4.7)	1.2 (0.84, 1.6)	10.9 (4.0)	1.1 (0.41, 2.7)	2.9 (3.4)
Rural health center – Type 2	2.3* (1.6, 3.4)	8.7 (8.0)	1.0 (0.58, 1.8)	8.7 (6.2)	0.74 (0.24, 2.3)	1.4 (1.7)
Rural health center – Type 1	1 (reference)	3.4 (2.4)	1 (reference)	9.2 (4.7)	1 (reference)	2.7 (2.5)

* *p*<0.05; N/A means this service does not exist at this factor/level (no patients observed).

## Discussion

In this time-motion study of over 8,000 patients across 12 health facilities in Central Mozambique, we found that healthcare wait times were long and consultation times were very short. Even the top 10th percentile of outpatient consultations did not last more than 10 min, and the lowest 10th percentile lasted 2 min or less. This contrasts with the top 10th percentile of waiting times for outpatient visits at around an hour and a half, and the lowest 10th percentile waiting 4 min. With an average outpatient consultation time of 5 min, this clearly is not sufficient to engage in all recommended primary healthcare tasks around patient history, vitals, physical examinations, risk factor counselling, and management regarding chronic conditions. These tasks do not even touch on the complexity of providing curative and preventative care for the health condition motivating the patient to come to the clinic in the first place.

Findings from the United States and Britain have indicated that longer visits are associated with a higher likelihood of hypertension screening, taking social or family history, consultation around cigarettes and alcohol, and preventative care (31). In one study in Britain, increasing appointment times from 5 min per patient to 7.5 min per patient was associated with identifying 50% more psychological problems and a 50% increase in blood pressure measurement (32). Given the central importance of high-quality outpatient primary care, antenatal care, and well-child consultations for ensuring the health of populations, increased efforts should be aimed at determining the optimal processes for patient flow and clinical activities in the context of stressed LMIC health systems.

One factor contributing to long wait times and bottlenecks in patient flow is the current situation whereby 48% of patients arrived to register at the clinic prior to 9:30 am and 71% before 10:30 am. Without the necessary administrative and coordination staff to manage patient appointments, lines for patient care often balloon in the morning hours. There are reports of patients waiting hours just to register at the clinic to begin queuing for their needed service. The large influx of patients in a short time period also creates problems for intrafacility referrals, specifically for outpatient consultations who often have to have lab tests and visit the pharmacy. While in the short term individual appointments may be difficult to operationalize, a move toward block scheduling or other innovative methods to spread patient loads out across the day could have a large effect on wait and consult times.

In our study, smaller rural facilities had shorter wait times, especially for first antenatal visits and well-child consultations. There was surprisingly large variability in mean consultation times for outpatient visits, with the smallest rural health centers having the longest consultation times and rural hospitals having almost twice the consultation time length compared with Type 1 rural health centers. These Type 1 rural health centers are large enough to have maternity, inpatient, laboratory, and pharmacy services but often have limited available technical staff and receive a steady flow of patients who cannot go to larger facilities often because of transport costs. A combination of few human resources and a relatively large amount of patients may cause the short consult times observed in the present study. Rural hospitals and larger referral hospitals (provincial and/or central hospitals) often have more technical staff and receive more complicated cases that may necessitate longer consultation times.

The factor most consistently associated with longer wait times for first antenatal visits and well-child visits was the number of institutional births – an association that held across health facility types. This is not surprising given that clinics having more institutional births are likely stressed with concomitant first antenatal visits prior to birth and well-child visits post-birth. This association may indicate that the distribution of maternal and child health (MCH) nurses within the public-sector health system is not optimized based on the number of institutional births conducted at clinics. Currently, provincial-level health departments allocate the number of MCH nurses to the district level. The district level then allocates nurses to specific health facilities based on facility level, type, and population need. In practice, however, the allocation of nurses is driven more by priority placement based on seniority or idiosyncratic factors, level of training, and specific local politics rather than an ideal allocation by facility burden or population health needs. Going forward, the HRH literature should prioritize further rigorous implementation science focused on optimizing sub-national management and allocation of limited trained health workers in LMICs.

The fact that those facilities servicing the largest number of expectant and post-partum mothers are also those that have the longest wait times is especially concerning given recent work showing that institutional birth attendance coverage is the health system factor most strongly associated with reductions in under-5, infant, and neonatal mortality in Mozambique (30). Second to institutional birth attendance, analyses have found that health workforce density is also strongly related to reductions in child death. While long wait times and short consult times are a management and coordination issue – improved patient flow and health system efficiency can only improve the current situation in as much as corresponding investments are made to increase public-sector HRH in Mozambique. Even with a 13% increase in health workforce density in Mozambique from 2000 to 2010 (30), Mozambique still ranks among the lowest countries worldwide in terms of physician or nurse density (33).

Furthermore, long wait times for the provision of MCH also has major implications for the expansion of HIV/AIDS treatment for pregnant women through Option B+. The implementation of Option B+ in Mozambique, which began in June–July of 2013, is expected to massively increase the workload of MCH nurses, likely resulting in substantially increased wait times. A recent formative research study to optimize Option B+ performance in public clinics in the same setting as the present time-motion study found that MCH nurses cited increased workload under Option B+ as patient volume increases as one of the largest challenges in their work (34). Lost to follow-up continues to be among the biggest challenges for successful PMTCT, and both long wait and short consultation times have been associated with lost to follow-up and poor treatment adherence (12, 13, 19, 35, 36). This challenge has been highlighted in Mozambique, with MCH nurses feeling understaffed, overworked, and underappreciated in the system leading to gaps in patient tracking, patient flow, long wait times, short consult times, and lack of follow-up of defaulters under Option B+ (34). Thus, these data drive home the urgency of training, recruiting, and retaining more maternal and child health nurses within the public-sector healthcare system to ensure the success of Option B+ in Mozambique. More generally, long wait times and short wait times should caution policymakers from rolling out new time-intensive treatment options or guidelines without a deep understanding of how these modifications may affect already stressed human resources and systems of primary health care.

In addition to considerations about optimizing health worker allocation and workload, additional health systems and operations research should be conducted in public-sector clinics to optimize: 1) tools and techniques used in consultations, diagnostics, and treatment; 2) protocols around patient flow management (37, 38); 3) supervision and data reporting; 4) role clarification and tasks of nurses, receptionists, counsellors, and other administrative staff (34); 5) data reporting requirements for routine data systems populated by already stressed clinic staff (39); 6) staff motivation and remuneration; and 7) allocation of scarce resources across geographic areas, clinics/hospitals, as well as disease-based health programs. In a recent systems analysis and improvement cluster-randomized trial among public clinics in Kenya, Cote D'Ivoire, and Mozambique, the majority of locally defined quality improvement micro-interventions focused on re-organizing services, clinic staff, and patient flow at the facility level – yielding significant improvements in ARV coverage and HIV-exposed infant screening (38).

The present study has a number of notable limitations. By including only 12 health facilities across two provinces (6% of 106 facilities in Manica and 4% of 156 facilities in Sofala), we cannot generalize across all health facilities of a given type or to other regions of Mozambique and were limited to bi-variate analyses around facility associated factors. In addition, we only assessed patient wait and consult times at one point during the year (January–April) and therefore this may not be representative of yearly utilization patterns. Given logistics around transport and lodging, we only began facility observations at 7:30 am. This could have had the effect of underestimating wait times since patients often begin queuing before normal business hours. We expect our estimates of consult times to be valid because we were present at facilities when they opened. Unfortunately, our group did not collect detailed staffing information for the 12 health facilities included in the present analyses in 2011. Thus, we were unable to conduct more detailed analyses around the relationship between patient loads per technical staff, among other potential analyses of interest. Since observation was conducted by our research teams on site, the Hawthorne (observer) effect may cause these data to not reflect the realities of daily service provision. Last, the present study did not assess quality of care directly, and this is an area that should be expanded on in future studies.

Our study also has a number of strengths. We observed a large number of patients across all levels of health service delivery in two provinces in Central Mozambique. Data on wait and consult times are based on direct observation rather than self-report, which improves reliability and validity of resultant data. Since data collection was conducted by the Beira Operations Research Center in partnership with the Provincial Health Directorate of Sofala Province, this partnership allowed these data to be rapidly and effectively used to guide future health system planning.

## Conclusions

Patients attending public-sector health clinics in Mozambique presently face unacceptably long wait times and short consult times. Intermediate-sized health facilities have significantly shorter outpatient consult times compared with larger hospitals or smaller rural health centres. The relatively strong association between the number of institutional births and wait times for first antenatal visits, and well-child visits may indicate that maternal and child health nurses are not optimally allocated according to facility burden. In the context of scaling-up Option B+ for PMTCT, current long wait times and short consult times for antenatal visits suggest that urgent investments must be made in MCH nurses to avoid poor treatment outcomes as a result of increased workload of already stressed human resources. Innovative operational and quality improvement studies, alongside continued investments in public-sector HRH are needed to increase consult times, decrease wait times, and improve health system responsiveness in Mozambique and other similar countries.

## Paper context

Mean wait times at primary care clinics in central Mozambique are unacceptably long, especially for first antenatal care visits. The average outpatient consult lasts only 5 min. Health facilities charged with providing maternal and child healthcare (MCH) to more of the population have the longest wait times for reception, first antenatal care visits, and well-child visits. This may indicate that the distribution of MCH nurses within the public health system is not currently optimized based on population need. Without urgent investment in public-sector human resources for health in Mozambique, increased workload for clinicians as new treatment and diagnostic norms are implemented for tuberculosis, malaria, HIV/AIDS, non-communicable diseases, and others may increase already long wait times and contribute to poor treatment effectiveness and low patient satisfaction.
